# The Complete Mitochondrial Genome of Eurasian Minnow (*Phoxinus* cf. *Phoxinus*) from the Heilongjiang River, and Its Phylogenetic Implications

**DOI:** 10.3390/ani12212960

**Published:** 2022-10-27

**Authors:** Lei Cheng, Ezhou Wang, Weitao Li, Xiaoli Yu, Xiaolin Liao

**Affiliations:** 1 Key Laboratory of Freshwater Aquatic Biotechnology and Breeding, Ministry of Agriculture and Rural Affairs, Heilongjiang River Fisheries Research Institute, Chinese Academy of Fishery Sciences, Harbin 150070, China; 2 Key Laboratory of Ecological Impacts of Hydraulic-projects, Restoration of Aquatic Ecosystem of Ministry of Water Resources, Institute of Hydroecology, Ministry of Water Resources and Chinese Academy of Sciences, Wuhan 430079, China

**Keywords:** mitochondrial DNA (mtDNA), Eurasian minnows (*Phoxinus* spp.), D-loop, phylogeny, Heilongjiang River

## Abstract

**Simple Summary:**

Fishes of genus *Phoxinus* belong to the family Leusciscidae and are widely distributed in Eurasia. Since the early 20th century, it has generally been accepted that the European minnow (*P. phoxinus*) was the only valid species of *Phoxinus* in Europe. After the loss of most species from North America and Asia, only those taxa in Euasia originally placed under the name *P. phoxinus* remain in the genus. In recent years, more than 20 lineages of genus *Phoxinus* have been identified in Europe, more than 10 of which have been considered as valid species in taxonomy. Existing morphological and genetic cues suggest distinct differences between Asian and European members of *Phoxinus*. Mitochondrial DNA from European *Phoxinus* spp. and their Asian relatives provide evidence that Far Eastern phoxinins should be divided into two genera: *Phoxinus* and *Rhynchocypris*. The updated phylogeny of *Phoxinus* indicates five mtDNA lineages in Asia. The validity of some of these lineages have been supported previously by morphological evidence.

**Abstract:**

Over the past two decades, the genus *Phoxinus* has undergone extensive taxonomic revision and many new species or mitochondrial lineages have been found in Europe. However, Asian populations of *Phoxinus* spp. have received less attention and have rarely been compared with their European relatives. In this study, we deciphered the 16,789-nucleotide mitochondrial genome of *Phoxinus* cf. *phoxinus* from the Heilongjiang River (HLJ) and compared it with other known mitogenomes or partial mitochondrial DNA (mtDNA) sequences of *Phoxinus* spp. We discovered that all known mitochondrial genomes of *Phoxinus* had a typical mtDNA architecture across vertebrates, but their D-loop regions varied greatly in length. A repetitive motif of ~130 bp was identified in the D-loop regions of *Phoxinus* spp. The unusual repetitive structure was revealed at the beginning of D-loop regions of all known mitogenomes of *Phoxinus* spp. The length differences of the D-loop region were attributed mainly to the number of repetitive motifs and the inserted sequences among them. However, this repetitive structure was absent in the other Far East phoxinins. This is further evidence for the notion that Far Eastern phoxinins should be divided into two genera: *Phoxinus* and *Rhynchocypris*. All mtDNA sequences (including three mitogenomes) from South Korea represent the same genetic lineage, as there were only slight differences among them. The remaining six mtDNA sequences are highly divergent and represent different lineages of the genus, as supported by partial mtDNA sequences. The updated phylogeny of genus *Phoxinus* suggests that there are five distinct mtDNA lineages in Asia. The Asian lineages have diverged markedly from their European relatives and should not be included with the European minnow (*P. phoxinus*).

## 1. Introduction

The fishes of genus *Phoxinus* belong to the family Leusciscidae and are widely distributed in Eurasia [1]. The classification of genus *Phoxinus* has changed dramatically over the past 20 years. The genus *Phoxinus* was once considered to be the only genus with a Holarctic distribution in suborder Cyprinoidei (the former Cyprinidae sensu lato), but molecular systematics suggests that North American species should not be attributed to genus *Phoxinus* [2]. The European minnow (*P. phoxinus*) has long been considered the only valid species of genus *Phoxinus* in Europe [3]. In recent years, a growing number of studies have shown that there are more than 10 valid species in the genus *Phoxinus* in Europe [3,4,5,6,7]. In Asia, most members of the Asian phoxinins should be reclassified as another genus, *Rhynchocypris*, with only “Asian populations” of European minnow (*P. phoxinus*) left in genus *Phoxinus* [8,9]. There are obvious differences in morphology and geographical distributions between Asian and European *Phoxinus* species, but Asian *Phoxinus* spp. are still mostly recognized as a subspecies or population of European minnow [1]. With the extensive revision of the taxonomy of *Phoxinus*, further clarification of the genus *Phoxinus* in Asia is urgently needed.

High phenotypic plasticity makes species delimitation of *Phoxinus* difficult and the wide distribution range makes comparisons between different types of minnows inconvenient [5,7,10,11,12,13,14]. Based on molecular data, a multispecies complex of *Phoxinus* in the Western Balkan Peninsula was reported and dispersion through subterranean water in karst formations was suggested [5]. Since this report, as many as 23 mtDNA lineages of genus *Phoxinus* have been reported [5,7,15,16,17,18]. Among the molecular markers used to study *Phoxinus*, the cytochrome oxidase subunit I (COI) gene encoded by mitochondrial DNA (mtDNA) seems to be the most successful [17,18] and has been generally used as a DNA barcode [19,20]. The application of molecular markers not only revealed substantial hidden diversity of *Phoxinus*, but also facilitated studies of geographical patterns, including invasion and diffusion patterns of the genus [18,21,22]. However, relevant studies on the molecular phylogeny and species delimitation of *Phoxinus* were mainly focused on European populations, and Asian populations have received less attention and have rarely been compared with European relatives.

Although there have been fewer comprehensive studies of *Phoxinus* spp. in Asia, the genetic structure of *Phoxinus* has been investigated with respect to their distribution [23,24,25,26,27,28,29] and mitochondrial DNA data have accumulated. Unfortunately, various mtDNA markers were used in these studies, and most of them were not COI genes, making direct comparison of different Asian populations or their European relatives difficult. The typical mtDNA of fish is a 16–17 kb circular molecule, characterized by a simple structure, maternal inheritance, lack of recombination and rapid evolution [30]. The complete sequence of the mitochondrial genome not only provides more genetic and evolutionary information than a partial sequence, but also provides the opportunity to integrate and compare different partial sequences. By searching GenBank and various publications, a total of 8 mitogenomic sequences of genus *Phoxinus* were retrieved, 2 from Europe and 6 from Asia [31,32,33,34]. By extracting the corresponding gene fragments from these known mitogenomes, previous results based on different fragments could be reanalyzed together. 

The primary purpose of this study was to explore the relationship between Asian populations of *Phoxinus* spp. and their European relatives. Six of 8 known mitogenomes were from South Korea, China and Mongolia, so there is a relatively wide geographical distribution. However, according to "*Fauna Sinica*", there are 3 subspecies of “*Phoxinus phoxinus*” in China [35]. The complete mitogenome of *Phoxinus (phoxinus) ujmoneesis* and *Phoxinus (phoxinus) tumenesis* have been determined [32,33]. In this study, the complete mitogenome of *Phoxinus* cf. *phoxinus* from the Heilongjiang River (HLJ) was successfully sequenced by an overlapping PCR method. Based on known mitogenomes of *Phoxinus* spp. and related studies, the phylogeny of *Phoxinus* was revised with special reference to species diversity in Asia.

## 2. Materials and Methods

### 2.1. Sample Collection and DNA Extraction

**Figure 1:**
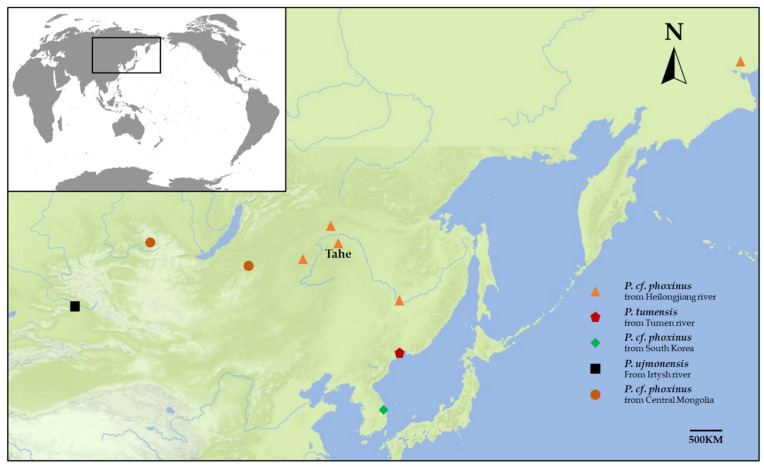
The sampling sites of Eurasian minnows (*Phoxinus* spp.) mitochondrial lineages in Asia. Triangles represent *Phoxinus* cf. *phoxinus* from the Heilongjiang River; Pentagon represents *P. tumensis* from Tumen River; Diamond represents the *Phoxinus* cf. *phoxinus* from South Korea; Solid circles represent *Phoxinus* cf. *phoxinus* from Mongolian Plateau; Square represents *P. ujmonensis* from Irtysh River. Samples from Anadyr River (in Northeast of Russia) clustered together with *Phoxinus* cf. *phoxinus* from HLJ, indicating that this lineage may have a wide distribution.

Specimens of *Phoxinus* cf. *phoxinus* from the Heilongjiang River (HLJ) were collected in Tahe County, Heilongjiang province, China (indicated in Figure 1 as Tahe). The *Phoxinus* cf. *phoxinus* samples were anesthetized before being preserved in 95% ethanol at room temperature. All animal procedures in this study were conducted according to the guidelines for the care and use of laboratory animals of Heilongjiang River Fisheries Research Institute, Chinese Academy of Fishery Sciences (CAFS). The studies in animals were reviewed and approved by the Committee for the Welfare and Ethics of Laboratory Animals of Heilongjiang River Fisheries Research Institute, CAFS. 

Total DNA was obtained from a fin clip of one individual using Proteinase K digestion and phenol-chloroform extraction. Some studies have reported complete or partial mitochondrial sequence of *Phoxinus* spp. from Asia and these publicly available mtDNA data were included in our analyses. Detailed information on the sequences used in this study are listed in Table 1, and the locations of *Phoxinus* populations in Asia are shown in Figure 1. The complete mitochondrial genome (X61010) of Common carp (*Cyprinus carpio*) was used as the outgroup [36].

### 2.2. Sequencing and Annotation of the Mitogenome

Based on the known mitogenomes of *Phoxinus* spp. and related species (Table 1), twelve primer pairs were designed to cover the complete mtDNA. When necessary, additional internal walking primers were designed for sequencing. Primer details were listed in Table S1 (Supplementary Materials). All primers were diluted to 10 μM, the PCR reaction volume was 30 μL and contained 1x PCRmix (Cowin bioscience, Beijing, China), 1 μL each of forward primer and reverse primer, and ~100 ng of template DNA. The amplification procedure was as follows: pre-denaturation at 94 °C for 2 min, followed by 35 denaturation cycles at 94 °C for 30 s; annealing at 58 °C for 30 s; extension at 72 °C for 1.5 min; final extension at 72 °C for 7 min. After amplification, the PCR products were tested using 1% agarose gel electrophoresis and commissioned to Sangon Biotech (Shanghai Co.; Ltd., Shanghai, China) for purification and sequencing.

The sequencing trace file was viewed on FinchTV v.1.3.1 (Geospiza, Inc., Seattle, WA, USA), and the low-quality clips at the beginning and end were trimmed. The mitogenome of HLJ were assembled with Cap3 [37] and submitted to MitoAnnotator (http://mitofish.aori.u-tokyo.ac.jp/annotation/input.html (accessed on 22 August 2022) for annotation [38]. Online software tRNAscan-SE 2.0 (http://trna.ucsc.edu/tRNAscan-SE (accessed on 22 August 2022) was used to locate the tRNA genes and analyze their secondary structure [39]. The D-loop region of known mitogenomes was analyzed by Tandem Repeat Finder software v. 4.07b to identify the repetitive units [40]. Then, repeated units were aligned with the ClustalX v.1.83 software [41] with manual modification to analyze their structure. The substructures of control regions were further analyzed, referring to the conserved domains of the Cypriniformes [42,43].

### 2.3. Phylogenetic Analysis

Mitochondrial lineages of *Phoxinus* were primarily defined by phylogeny based on COI sequences. Because of several prior barcoding projects and phylogenetic studies [5,7,15,16,17,18], COI sequences were available for all but lineage 21 [16]. To begin, COI sequences of known mitogenomes were extracted for comparison with available COI sequences of *Phoxinus* from across Europe [18]. Several *Cyt b* and 16S rRNA sequences of Asian *Phoxinus* were available in previous publications [9,24,25,26,27,28,29], so *Cyt b* and 16S rRNA sequences of known mtDNA were extracted together for analysis. Thus, three different datasets were subjected to phylogenetic analysis: (1) cytochrome oxidase subunit I (COI) sequences; (2) Cytochrome b (*Cyt b*) sequences; and (3) 16S rRNA sequences (Table 1). Following previous studies [29], *Rhynchocypris lagowskii* (AP009147) was chosen as the outgroup.

Sequences were aligned with MUSCLE [44], and alignments were verified visually. Phylogeny reconstruction was performed using Bayesian inference (BI) and maximum-likelihood (ML) approaches. The best-fit nucleotide substitution models of Bayesian inference (BI) were selected by mrModeltest v2.4 [45] using the Hierarchical Likelihood Ratio Tests (hLRTs). Bayesian analysis was conducted using MrBayes v3.2 [46]. Four chains were run for 5,000,000 generations, sampling trees every 100 generations and the first 12,500 trees were discarded as burn-in. The ML analysis was conducted by combining ModelFinder, tree search, SH-aLRT test and ultrafast bootstrap with 1000 replicates in IQ-TREE [47]. 

## 3. Results and Discussion

### 3.1. Characterization of the Mitogenome

The complete mitochondrial genome of *Phoxinus* cf. *phoxinus* (HLJ) was 16,789 bp, with an overall GC content of 43.79%, and it has been deposited into GenBank under accession OP326577. Our annotation identified 2 rRNAs (12S and 16S rRNA), 22 tRNAs, 13 protein-coding genes (PCGs) and 1 D-loop region, which were consistent with the gene content of typical mtDNA in the teleost. As shown in Table 2, the arrangement of genes was quite compact. There were 14 spacers between genes or elements, ranging from 1 to 33 bp, totaling 64 bp. There were 10 overlapping regions, with 1−7 bp shared bases, totaling 28 bp. Excluding the D-loop region, the longest non-coding sequence is the origin of light strand replication (O_L_). As is typical with the teleost, only 1 protein-coding gene (ND6) and 8 tRNA genes are encoded by the light strand (L-strand) and all other genes are encoded by the heavy strand (H-strand). Compared with the gene order of typical vertebrate mtDNA, no rearrangement was observed in the mtDNA of *Phoxinus* cf. *phoxinus* (HLJ).

Most (11/13) of the protein-coding genes (PCGs) started with ATG, while the starting codons of COI and ND3 genes are GTG. The stop codons of 10 PCGs are complete, of which 5 stop codons are TAA (COI, ATP6, ND4, ND5, ND6) and 5 stop codons are TAG (ND1, ND2, ATP8, ND3, ND4). The stop codons of the remaining 3 PCGs are incomplete, of which 1 PCG has a stop codon for TA-(COII), and the other 2 PCGs have stop codons for T- (COIII, *Cyt b*), and post-transcriptional modifications are required to form a complete stop codon in these 3 PCGs. There are 4 overlapping regions between PCGs, with 7 bp between ATP8 and ATP6, 1 bp between ATP6 and COIII.; 7 bp between ND4L and ND4, and 4 bp between ND5 and ND6.

Two ribosomal RNA (rRNA) genes of the HLJ mitogenome are similar to their counterparts in other fish. The 12S rRNA was 954 bp, located between tRNA-Phe and tRNA-Val. The 16S rRNA gene was 1682 bp, located between tRNA-Val and tRNA-Leu (UUR). Twenty-two tRNA genes of the HLJ mitogenome ranged from 69 bp to 76 bp, transporting a total of 20 amino acids. With the exception of tRNA-Ser (UAG), all tRNA genes can be folded into cloverleaf secondary structures. Of these 22 tRNAs, 14 were encoded on the H-strand and the remaining 8 (tRNA-Gln, tRNA-Ala, tRNA-Asn, tRNA-Cys, tRNA-Tyr, tRNA-Ser (UCN), tRNA-Glu, and tRNA-Pro) were encoded on the L-strand. All of the features described above are similar to the general features of the mitochondrial genome of cyprinids.

### 3.2. The D-loop Region of Phoxinus Spp.

**Figure 2:**
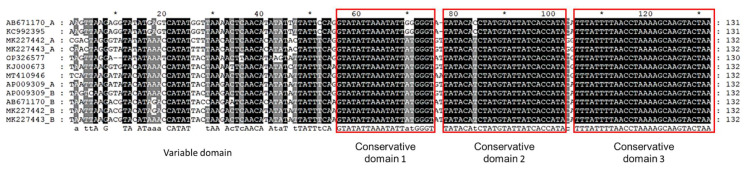
Aligned~130bp repetitive motif to show the conservative domain (in red box) and variable domain. The number on the right indicates the length of each repetitive motif. The number above indicates the position of the corresponding nucleotides. The sign “*” between the two numbers indicates the midpoint between them.

Like other cyprinids, the D-Loop regions of *Phoxinus* spp. are located between tRNA-Pro and tRNA-Phe, but their length has a larger variation, from 991 bp (MT410946) to 2544 bp (MK227443) in genus *Phoxinus*. Excluding the D-loop region, mitochondrial genomes of *Phoxinus* spp. were highly conserved in length. We found a 130 bp repetitive unit at the beginning of the D-loop of *Phoxinus* cf. *phoxinus* (HLJ) and there are similar repetitive structures in the known mtDNA of the genus *Phoxinus* (Figure 2). Each repetitive unit can be further divided into a variable domain and 3 conservative domains. Our comparative analysis suggests that the length variation of the D-loop region is directly related to the number of repetitive units and length of the inserted sequences between the repetitive units. The longest D-loop region was found in MK227443, in which the repetitive unit appeared up to 6 times, and partial sequences of D-loop regions were also inserted between some repetitive units. However, there was only 1 copy of a repetitive unit without insertion of other sequence in the shortest D-loop region (MT410946). We speculate that the repetitive structure may have mediated the insertion and reorganization of fragments between repetitive units.

**Figure 3:**
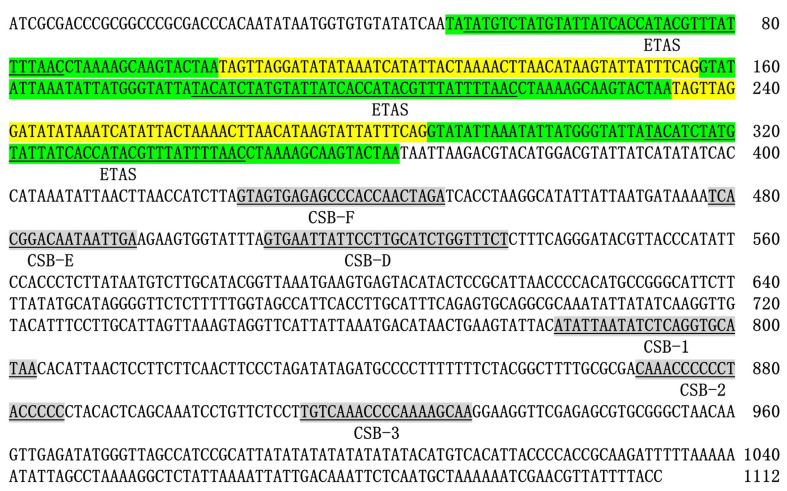
D-loop of *Phoxinus* cf. *phoxinus* from the Heilongjiang River, with special reference to important regulatory elements that are underlined and shaded. Conservative domain and variable domain of repetitive unit are shown in green and yellow.

As shown in Figure 3, several important regulatory elements of cyprinids were present in the D-loop of *Phoxinus* cf. *phoxinus* (HLJ). The extended termination associated sequence (ETAS) domain was identified at the 5’ end of the control region. In cypriniforms, the consensus sequence of ETAS is TACAT---ATGTATTATCACCA---TATTTAACCATAAA [42], similar to the conservative domain of the repeat motif. Extended termination associated sequence (ETAS) domain acts as a signal for termination of H-strand synthesis. Repeated termination signals have also been found in other vertebrates [48,49], but it seems to be that the first ETAS plays a major role [50]. This point was also supported by the D-loop structure of *Phoxinus* spp. At the beginning of the D-loop, there is an independent ETAS at the front of repetitive region. The sequence after the repetitive region was similar to the homologous sequence of other cyprinids. CSB-D and CSB-E have also been identified in the central domain, but a sequence (GTAGTGAGAGCCCACCAACTAGA) in HLJ shown limited similarity with CSB-F identified in other cyprinids [42]. All three elements of conserved sequence blocks (CSB1-3) were identifiable in the 3′ end of the control region [42,43]. The base composition of the D-loop region of HLJ also has a significant A+T bias (66.5%) and is greater than the A+T content of other regions of the mitochondrial genome.

### 3.3. Phylogeny of Phoxinus Spp.

**Figure 4:**
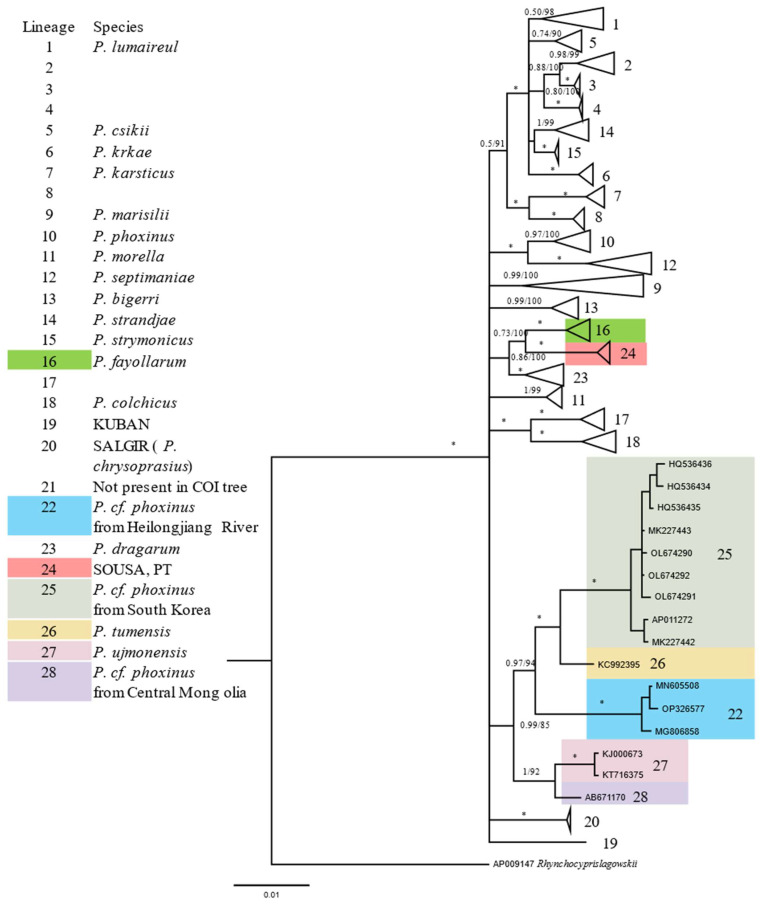
A Bayesian inference (BI) tree of Eurasian *Phoxinus* based on cytochrome oxidase I (COI) data. Posterior probability values for BI (BPP) and bootstrap values for maximum-likelihood (ML) are shown on branches. The sign ‘’*‘’ indicated BPP of 1 and Bootstrap values of 100. Genetic lineages are presented in the upper left corner. The genetic lineages that are valid species are written on the right. Only newly added or modified lineages are marked in color. Asian clades were expanded to show the sequence contained in each lineage more clearly. *Rhynchocypris lagowskii* (AP009147) was used as the outgroup.

We added the COI gene sequences from Table 1 into the COI dataset of Palandačić et al. [18] for phylogenetic analysis. The results obtained by the two methods (BI and ML) are highly consistent. The major difference between our results and those of previous studies is the number of identified clades. In previous studies, 22 linages of *Phoxinus* were identified based on the COI dataset [18], and an additional lineage 21 was identified based the *Cyt b* data [16,17,18]. In this study, we identified 27 linages from the COI dataset (also excluding lineage 21) by involving the COI sequences of Asian *Phoxinus* spp. The 5 additional clades are from South Korea, Tumen River, Irtysh River, Mongolian Plateau and Portugal. To avoid confusion, we numbered these 5 lineages following previous studies (Figure 4). The mtDNA of HLJ was clustered with lineage 22 from the previous study. A newly defined lineage 24 was also presented in previous studies, which had previously been classified with lineage 16 [18], rather than an independent clade. Of the COI fragments extracted from the whole mitogenome sequences, three from South Korea form one lineage (numbered 25) [26,34]. However, each of the remaining 5 COI sequences represented a distinctive mitochondrial lineage. AP009309 and AP009147 belong to lineage 1 and 11, respectively, and the other 3 sequences from Asia correspond to 3 new lineages (linages 26–28).

**Figure 5:**
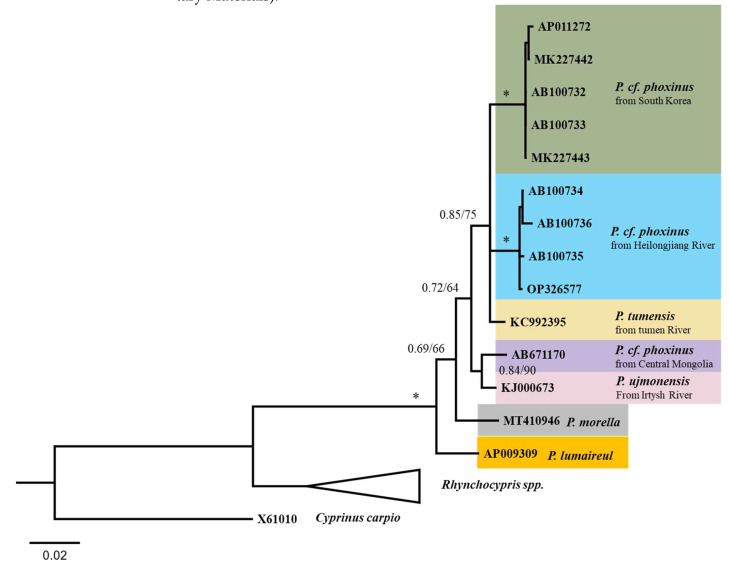
A Bayesian inference (BI) tree of Far Eastern phoxinins based on mtDNA 16S rRNA gene data. Posterior probability values for BI (BPP) and bootstrap values for maximum-likelihood (ML) are shown on branches. The sign ‘’*‘’ indicated BPP of 1 and Bootstrap values of 100. The 16S rRNA sequence (X61010) of Common carp (*Cyprinus carpio*) was used as the outgroup.

Based on allozyme and mitochondrial 16S rRNA sequences, Sakai et al. [9] concluded that Far Eastern phoxinins should be split into two genera: *Phoxinus* and *Rhynchocypris*. The corresponding 16S rRNA sequences of 9 mitochondrial genomes were extracted and added to the dataset of Sakai et al. [9]. Reanalyzed results were highly consistent with that of Sakai et al. [9], showing that the separation of *Phoxinus* and *Rhynchocypris* was primary (Figure 5). The results from different datasets are consistent and indicate that all *Phoxinus* samples from South Korea represent the same mitochondrial lineage (see in Figure 4, Figure 5 and Figure S1). All mtDNA sequences of *Phoxinus.* cf. *phoxinus* from HLJ cluster into the same mitochondrial lineage 22. Interestingly, samples from the Anadyr River (in Northeast Russia) also cluster into Lineage 22, indicating that this lineage may have a wide distribution. *Phoxinus* spp. from the Irtysh River, Mongolian Plateau and Tumen River, each representing a distinct lineage, were not included in previous data of Sakai et al. The results of *Cyt b* data analysis were consistent with those of COI and 16S rRNA (Figure S1 in Supplementary Materials).

Kottelat studied the morphology of *Phoxinus* spp. from Mongolia and argued that they were markedly different from European minnow (*Phoxinus phoxinus*) [1]. He also pointed out that *Phoxinus* spp. from the Selenge, Kherlen and Bulgan drainages differed from each other, and suggested that they were distinct species [3]. Our results support the existence of 3 lineages in Mongolia. Lineage 23 from the central Mongolia Plateau was collected at Ulaanbaatar [29] and the Tyva Republic [28]. Eastern samples from the Kherlen River (a tributary of the HLJ) belong to Lineage 22. Populations from western Mongolia are speculated to be conspecific with Altai populations and discussed under *P. ujmonensis* (Irtysh River) [1]. In a phylogenetic analysis, the mtDNA of *P. ujmonensis* also formed an independent lineage (No. 27). "*Fauna Sinica*" recorded that there were 3 subspecies of “*Phoxinus phoxinus*” in China, two are the above-mentioned *P. ujmonensis* from Irtysh River and *Phoxinus* cf. *phoxinus* from HLJ, and the third is *P. tumenesis*, which has a complete lateral line and is distributed only in the Tumen River Basin. The mtDNA of *P. tumenesis* also formed a distinct lineage (No. 26). All mtDNA sequences of *Phoxinus* cf. *phoxinus* from South Korea cluster into a distinct lineage, though it is closely related to *P. tumenesis*. Thus, there are a total of 5 Asian lineages of *Phoxinus* now identified. They are significantly different from their European relatives, suggesting that Asian populations of *Phoxinus* should no longer be identified as “*Phoxinus phoxinus*”.

## 4. Conclusions

In this study, we deciphered the complete mitochondrial genome of *Phoxinus* cf. *phoxinus* from the Heilongjiang River and the results were analyzed and compared with other mtDNA datasets. An unusual repetitive structure was revealed at the beginning of D-loop regions of all known mitogenomes of *Phoxinus* spp. However, this repetitive structure was absent in phoxinin populations that we propose reclassifying as *Rhynchocypris* spp. This is further evidence for the phylogenetic result that Far Eastern phoxinins should be divided into two genera: *Phoxinus* and *Rhynchocypris*. The updated phylogeny of *Phoxinus* also indicates 5 mtDNA lineages of *Phoxinus* in Asia that are deeply divergent from their European relatives. Northern Asia is suitable for the distribution of *Phoxinus* species, but there are many areas not covered in previous publications. Given the tremendous progress made in taxonomy and the phylogeography of *Phoxinus* in Europe, there is no doubt that more lineages or species of *Phoxinus* may be found in Northern Asia.

## Figures and Tables

**Table 1:** The samples used for phylogenetic analysis and accession numbers of their mtDNA sequences.

Species/Lineages	Mitogenomes	COI	*Cyt b*	16s rRNA	Sample Size	Reference
*P. cf. phoxinus*	OP326577				1	this study
(Heilongjiang River)		MG806858	MG806682		1	[27]
		MN605508			1	[28]
				AB100734-AB100736	44	[9]
*P. tumensis*	KC992395				1	[32]
*P. ujmonensis*	KJ000673				1	[33]
			KR819892-KR819906		15	[25]
		KT716375			1	[24]
*P. cf. phoxinus*	AB671170				1	[31]
(Central Mongolia)			MW001939-MW001940		15	[29]
*P. cf. phoxinus*	AP011272				1	[31]
(South Korea)	MK227442				1	[34]
	MK227443				1	[34]
			KX265376-KX265402		56	[26]
				AB100732-AB100733	40	[9]
*P. lumaireul*	AP009309				1	[31]
*P. morella* ^1^	MT410946				N/A	Unpublished
*Rhynchocypris lagowskii*	AP009147				1	[31]
*Rhynchocypris* spp.				AB100697-AB100731	582	[9]
*Cyprinus carpio*	X61010			X61010	1	[36]

^1^ There are two mitogenomes from the same isolate DM856 of Bleak (*Alburnus alburnus*) in Genbank, which were submitted by the same author (Leerhoei, F.) on 29-APR-2020 and 09-JUN-2020, respectively. One (MT584105) is highly similar to Bleak mitochondrial reference genome, but the other (MT410946) should come from *Phoxinus morella* based on our analysis. This sequence (MT410946) was the only unpublished one involved in our study.

**Table 2:** Organization of the mitochondrial genome of *Phoxinus* cf. *phoxinus* from the Heilongjiang River (HLJ).

Gene/Element	From	to	Length(bp)	Start	Stop	Intergenic Nucleotides	Coding Strand
tRNA-Phe	1	69	69				
12S rRNA	70	1026	957				
tRNA-Val	1027	1098	72				
16S rRNA	1099	2790	1692				
tRNA-Leu (UAA)	2791	2866	76				
ND1	2868	3842	975	ATG	TAG	1	
tRNA-Ile	3847	3918	72			4	
tRNA-Gln	3917	3987	71			−2	L
tRNA-Met	3989	4057	69			1	
ND2	4058	5104	1047	ATG	TAG		
tRNA-Trp	5103	5173	71			−2	
tRNA-Ala	5175	5243	69			1	L
tRNA-Asn	5245	5317	73			1	L
OL	5318	5348	31				
tRNA-Cys	5349	5416	68				L
tRNA-Tyr	5418	5488	71			1	L
COI	5490	7040	1551	GTG	TAA	1	
tRNA-Ser (UGA)	7041	7111	71				L
tRNA-Asp	7114	7187	74			2	
COII	7202	7892	691	ATG	T--	14	
tRNA-Lys	7893	7968	76				
ATPase 8	7970	8134	165	ATG	TAG	1	
ATPase 6	8128	8811	684	ATG	TAA	−7	
COIII	8811	9595	785	ATG	TA-	−1	
tRNA-Gly	9595	9665	71			−1	
ND3	9666	10016	351	GTG	TAG		
tRNA-Arg	10015	10083	69			−2	
ND4L	10084	10380	297	ATG	TAA		
ND4	10374	11756	1383	ATG	TAG	−7	
tRNA-His	11756	11824	69			−1	
tRNA-Ser (GCU)	11825	11893	69				
tRNA-Leu (UAG)	11895	11967	73			1	
ND5	11968	13803	1836	ATG	TAA		
ND6	13800	14321	522	ATG	TAA	−4	L
tRNA-Glu	14322	14390	69				L
*Cyt b*	14396	15536	1141	ATG	T--	5	
tRNA-Thr	15537	15608	72				
tRNA-Pro	15608	15677	70			−1	L
control region	15678	16789	1112				

## Data Availability

The complete mitochondrial genome of *Phoxinus* cf. *phoxinus* from the Heilongjiang River has been deposited in GenBank under accession OP326577. All other sequences used in this study can be downloaded from GenBank with accession numbers listed in Table 1 and reference [18].

## Supplementary Materials

The following supporting information can be downloaded at: https://www.mdpi.com/article/10.3390/ani12212960/s1, Figure S1: Bayesian inference (BI) tree of Eurasian *Phoxinus* based on Cytochrome b (*Cyt b*) data; Table S1: PCR and internal walking primers for sequencing the mitogenome of *Phoxinus* cf. *phoxinus* from the Heilongjiang River (HLJ).
