# Supplementary material for: The Complete Mitochondrial Genome of Eurasian Minnow (Phoxinus cf. Phoxinus) from the Heilongjiang River, and Its Phylogenetic Implications
Source: Animals (Basel). 2022 Oct 27;12(21):2960. doi: 10.3390/ani12212960 (PMC9658870; doi:10.3390/ani12212960)
Supplement: Supplementary file 1 [file animals-12-02960-s001.zip › animals-1933492-supplementary.pdf]

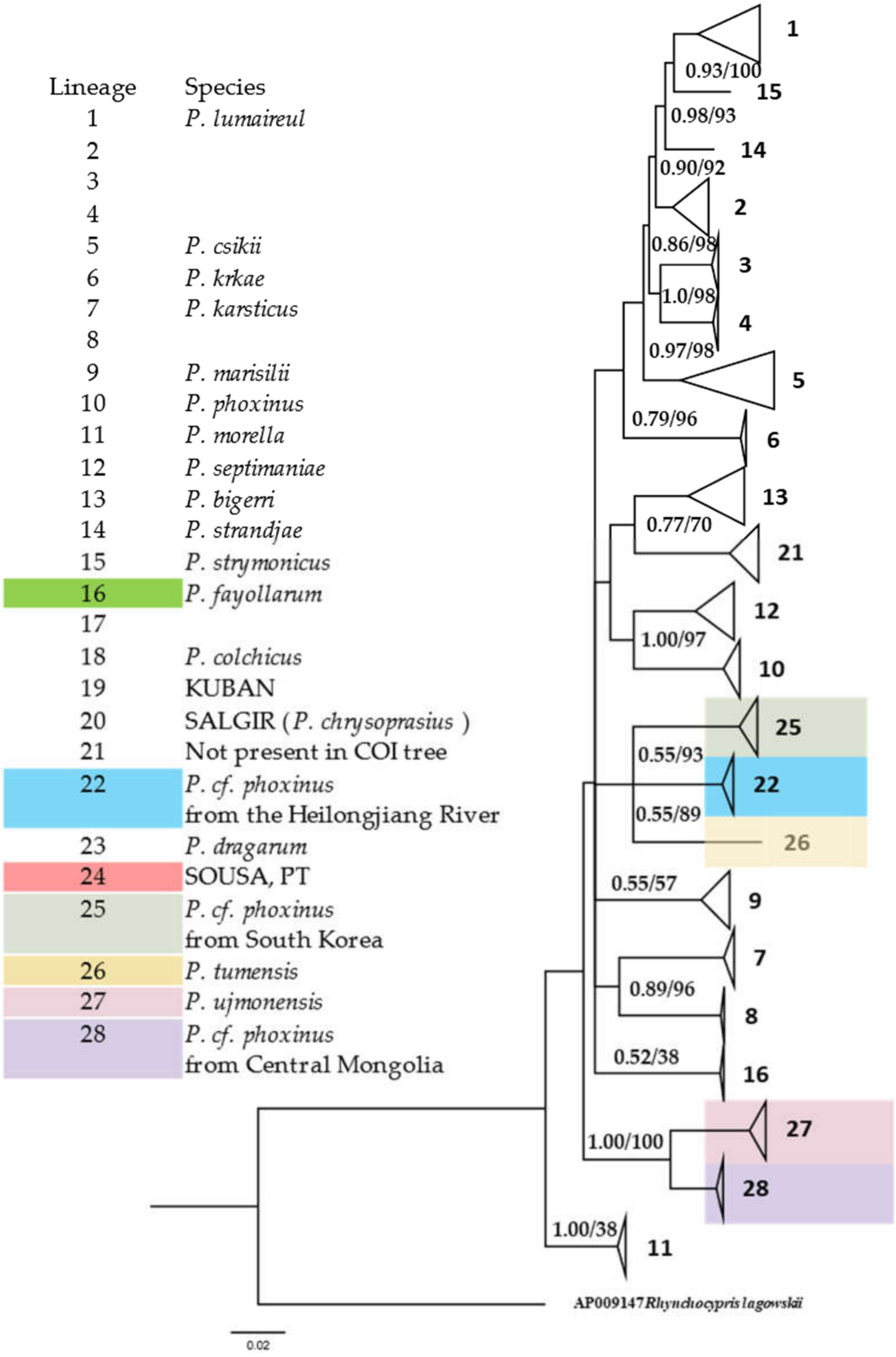

Figure S1. Bayesian inference (BI) tree of Eurasian *Phoxinus* based on Cytochrome b (*Cyt b*) data. Posterior probability values for BI (BPP) and bootstrap values for maximum-likelihood (ML) are shown on branches. Genetic lineages are presented in the upper left corner. The genetic lineages that are valid species are written on the right. Only Asian clades lineages are marked in color. *Rhynchocypris lagowskii* (AP009147) was used as the outgroup.

Table S1. PCR and internal walking primers for sequencing the mitogenome of *Phoxinus* cf. *phoxinus* from the Heilongjiang River (HLJ).

Primers	Sequence (5′-3′)	Primers	Sequence (5′-3′)
PCR01F	GTTGGATCAGGACATCCTAATGGTGCA	PCR01R	AGAAAGTGGTGTAGAGGAAGC
PCR02F	CCTAAGGACCACTTTGATAGAGTG	PCR02R	GCCACAGGTAGGGTAGCYGAGT
PCR03F	CCAAGCCAGCGAGCATCYATCTAC	PCR03R	CTGGCTTGAAACCAGCATATGG
PCR04F	GCCTACGCCCTGTGAAATACAGT	PCR04R	CCAGATTGCTAGRCCGGATGT
walking4	GATAGGACAYCAATGATACTG		
PCR05F	ATGGCCCACCAAGCACATGCATATC	PCR05R	GAGATTAAGGTTTTGTAGACGGTC
PCR06F	GACATTTCAGCTTTAGCTCAGC	PCR06R	GCACCAAGAGTTTTTGGTTCCTAAGACC
PCR07F	GACATTAGATTGTGATTCTAA	PCR07R	GTGGAGGAATGCTAGTTGTGGTT
PCR08F	GGAAAGTCAGCCCAATTTGGCC	PCR08R	GGGTCTTTCGTAGGCTTGCCAT
walking8	CTACTCTAAAACTMGCAGCCCT		
PCR09F	GGAGTAGGATTAGAAGCAACAGC	PCR09R	GCTTTGGGAGYCAGGGGTGRGAGTT
PCR10F	GAGCTTGCACTAGTAGCTTAG	PCR10R	GGTAACGTATCCCTGAAAGAG
PCR11F	AGTAGTGAGAGCCCACCAAC	PCR11R	GGACTCCTAGAAGCGTATGAC
PCR12F	GGTAAAACTCGTGCCAGCCA	PCR12R	GCCCCTCTTTTCCGRTCCTTTCGTACTA
walking12	GGCATCTCACTTACACCGAGAA		
